# Synergistic Effect of Vitamin C with Cisplatin for Inhibiting Proliferation of Gastric Cancer Cells

**DOI:** 10.29252/ibj.24.2.119

**Published:** 2019-10-29

**Authors:** Ghazaleh Ghavami, Soroush Sardari

**Affiliations:** Drug Design and Bioinformatics Unit, Medical Biotechnology Department, Biotechnology Research Center, Pasteur Institute of Iran, Tehran, Iran

**Keywords:** Ascorbic acid, Cisplatin, Neoplasms

## Abstract

**Background:**

Ascorbic acid, known as vitamin C, has been used in combination with a number of cytotoxic agents *in vitro* and *in vivo* with contradictory results on its effectiveness. It is believed that vitamin C can sensitize different cancer cells to common therapy strategies such as chemotherapy and radiotherapy. During current research, the combination effect of vitamin C with cisplatin was evaluated against gastric cancer cells.

**Methods:**

MTT-based proliferation assay, CI method, and flow cytometry technique were employed for the assessment of cell cycle and determination of apoptosis/necrosis on the AGS cell line.

**Results:**

Co-treatment of gastric cancer cells with vitamin C in its IC_50_ dose in addition to cisplatin in both IC_50 _(10 µg/ml) and five times less (2 µg/ml) doses could increase the cytotoxicity effect of cisplatin in a synergistic manner. Moreover, the pointed co-treatment approach could induce the cell count in sub-G0 phase while reducing it in the G0/G1, G2/M, and S phases. Further findings showed that the combined dose of vitamin C and cisplatin could increase the percentage of apoptotic and necrotic cells in comparison with a single dose of cisplatin.

**Conclusion:**

This study introduces a possible approach for the treatment of gastric cancer with more potency and less amount of administered cisplatin to induce toxicity.

## INTRODUCTION

Ascorbic acid, commonly known as vitamin C, is utilized as one of the most important dietary supplements and safe medicines in the health system based on the reports of World Health Organization's Model List of Essential Medicines^[^^1^^]^. This vital antioxidant supplement plays critical roles in the process of repairing tissues, producing neurotransmitters through enzymatic mechanisms, and modulating of immune system. It is also known as the most powerful radical scavenger agent^[^^1^^,^^2^^]^. 

 Herbs containing vitamin C have been reported to be from families of Rutaceae (*Citrus *sp.), Actinidiaceae (*Actinidia *sp.), Brassicaceae (*Brassica *sp.), Solanaceae (*Capsicum *sp.), Rosaceae (Fragaria *sp.)*^[^^1^^]^*,* Moraceae (*Morus *sp., specially *Morus nigra* and *Morus alba*), Musaceae (*Musa *sp., particularly *Musa paradisiaca* and *Musa sapientum*)^[^^3^^-^^5^^]^. 

 Findings show that vitamin C, as one of the first major defense systems against aqueous radicals in blood, can have a critical role in protecting biological membranes against primary peroxidative damages. In addition, it has ability to reduce oxygen, nitrogen, and sulfur radicals in its non-toxic doses^[^^2^^]^. In fact, vitamin C can reduce nitrate and prevent the generation process of carcinogenic nitrosamines by employing NADH-dependent system^[^^2^^]^. 

 The growth inhibitory properties of vitamin C and its derivatives in more than seven varieties of cancer cells without inducing cytotoxic effects on normal fibroblast cells have previously been indicated^[^^2^^]^. It has also been demonstrated that the cytotoxic effect of vitamin C on a set of malignant cell lines is commonly related to its pro-oxidant property, which leads to the activation of transcription factor NF-kappa B and finally, the cell growth inhibition^[^^2^^]^. Furthermore, with generating hydrogen peroxide and activating hydroxyl radical, vitamin C can reduce cell replication mechanism as well as tumor cell viability via inducing DNA strand breakage, controlling mitotic activity, and damaging the mitochondria and cell membranes. Vitamin C has also been found to act as an anticancer agent by reduction in hypoxia-inducible factor 1, causing reductive energy, i.e. vitamin C can induce tumor cell death via stabilizing p53, as a main protein involved in controlling cell proliferation^[^^2^^]^. Reported data have suggested that the administration of vitamin C alone can induce toxicity on AGS cell line through the activation of caspase cascades and the related apoptotic pathway. This activation may occur by inducing calcium efflux from endoplasmic reticulum, generating reactive oxygen species, reducing adenosine triphosphate production, and stimulating autophagy pathway^[^^6^^,^^7^^]^. 

 Further relevant studies have revealed that combining vitamin C with 5-fluorouracil, sodium d-ascorbate, cyclophosphamide, paclitaxel, arsenic trioxide, doxorubicin, and radiation can sensitize the cancer cells to the exposed anticancer drugs and enhance the success rate of hemo/radiotherapy^[^^8^^,^^9^^]^. Indeed, the clinical evaluation confirmed the major positive effect of vitamin C on enhancing the quality of life in 30–95% of cancer patients during chemo/radiotherapy procedures^[^^9^^]^.

 The current investigation was attempted to study the combination treatment effect of vitamin C with cisplatin on gastric cancer cells, in order to introduce a new possible treatment strategy of gastric cancer with more efficacy parallel to the reduction of cisplatin side effects.

## MATERIALS AND METHODS

Cell culture 

 Human gastric adenocarcinoma (AGS) cell line (IBRC C10071) was purchased from *Iranian Biological Resource Center* (Tehran, Iran) and cultured in RPMI 1640 (Biosera, France) with 10% fetal bovine serum and 2 g/l of HEPES buffer (both from Biosera) in 5% CO_2_ at 37 °C. The cells with 80% confluency were passaged by Trypsin-EDTA solution 1 (Biosera). The 96- and 24-multiwell plates were used to perform MTT and cell cycle/apoptosis tests for seeding the cells at a density of 10^4^ cells/cm^2^ and 10^5^ cells/cm^2^*,* *respectively.*

Cell proliferation assay

 AGS cells were seeded on 96‐well plates, and the medium was replaced with serum‐free RPMI 1640 medium containing vitamin C (Sigma-Aldrich, Germany) in 8, 40, 200, and 1000 μg/ml and cisplatin (Mylan, Netherlands) in 0.8, 4, 20, 100, and 500 μg/ml doses as the positive control. Serum‐free RPMI 1640 medium was used as the solvent and negative control. The treatment process was performed in 5% CO_2_ at 37 °C for 48 hours. After the treatment, each well received 20 μl of MTT dye (0.5 mg/mL; Sigma-Aldrich) was added to each well, and the plate was incubated at 37 °C for 4 hours. Subsequently, the treatment medium was replaced by DMSO (Fluka, USA). After 30 min, the absorbance level of each well was measured with a multi-well scanning spectrophotometer (ELISA reader, Organon Tekninka, Netherlands) at the wavelength of 545 nm. The results were calculated as cell toxicity rate = 1 - (OD of the sample well-OD of the blank well)/(OD of control well-OD of blank well) × 100%^[10]^. All the measurements were conducted in triplicates. 

**Assessment of the cell cycle progression**

 For cell cycle analyses, AGS cells (1.0 × 10^5^ cells/cm^2^) were treated with vitamin C alone and in combination with cisplatin for 48 hour. The treated cells were then fixed with 70% ethanol in cold room for 5 hour and then stained with 0.1% Triton X-100 (Sigma-Aldrich), 0.5 mg/ml of ribonuclease A (Sinaclon, Iran), and 0.025 mg/ml of PI (Sigma-Aldrich) at room temperature for 30 min before flow cytometry by CyFlow (Partec, Germany)^[^^11^^]^. 

Apoptosis and necrosis detection

 The rate of apoptosis was investigated by annexin-V and PI double staining^[^^12^^]^. After the treatment of AGS cells (1.0 × 10^5^ cells/cm^2^) by vitamin C alone and in combination with cisplatin for 48 hour, the cells were pooled and centrifuged at 112 RCF at 4 °C for 5 min. Next, the treated cells were stained with annexin V-fluorescein isothiocyanate and PI (IQ Products BV, Netherlands) based on the manufacturer's procedure and analyzed by a flow cytometer (CyFlow).

***Statistical analysis***

 Entire tests were performed as triplicate tests (n = 3), and relevant results were analyzed by Graph Pad Prism 5.0 software (GraphPad, La Jolla, CA, USA) to calculate IC_50_s (regression equation with the 95% confidence interval) and mean ± SEM. Statistical comparisons were conducted by ANOVA and *post hoc* Tukey's test utilizing Graph Pad Prism 4.0 and Statistical 6.0 program. The differences were considered as statistically significant when p < 0.05. CI method was employed to evaluate the interaction between vitamin C and cisplatin. CI was presented by ComboSyn (ComboSyn Inc., NY, USA) as the CI theorem of Chou-Talalay, which is a quantitative assessment of expressions of synergism (CI < 1), additive (CI *=* 1), and antagonism (CI *>*1).

**Fig. 1:**
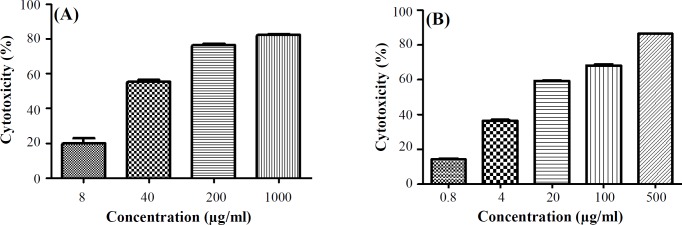
The cytotoxic effects of (A) vitamin C (49.38 ± 0.82 µg/ml) and (B) cisplatin (10.58 ± 0.49 µg/ml) alone on AGS cell line. Data are shown as mean ± SEM. Statistical comparison with solvent (p < 0.05) was calculated for vitamin C and cisplatin as *p* < 0.001

## RESULTS

**Cell proliferation assay**

**Fig. 2:**
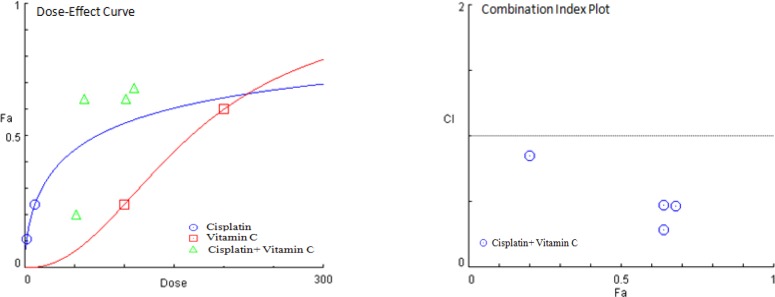
CI method for the assessment of the combination effects of vitamin C with cisplatin on gastric cancer cells. CI plot presents CI at different ratios of the Fa. Calculated data demonstrated the synergism (CI < 1) between vitamin C and cisplatin

 MTT assay was performed to study the cytotoxic properties of vitamin C alone (Fig. 1) and in combination with cisplatin *(*Fig. 2* and *Table 1*) *on AGS cell line. In dose of 49.38 ± 0.82 µg/ml, vitamin C showed cytotoxic effect on AGS cells. Evaluating the combined cytotoxic effects of vitamin C with cisplatin on gastric cancer cell line was carried out based on CI theorem of Chou-Talalay*. Treatment of AGS cells with the combined dose of *vitamin C and cisplatin was led to higher *in vitro* cytotoxic effect of cisplatin against gastric cancer cells in both IC_50 _and five times less than IC_50 _doses in a synergistic manner with CIs < 1.

**Cell cycle progression assay**

**Fig. 3:**
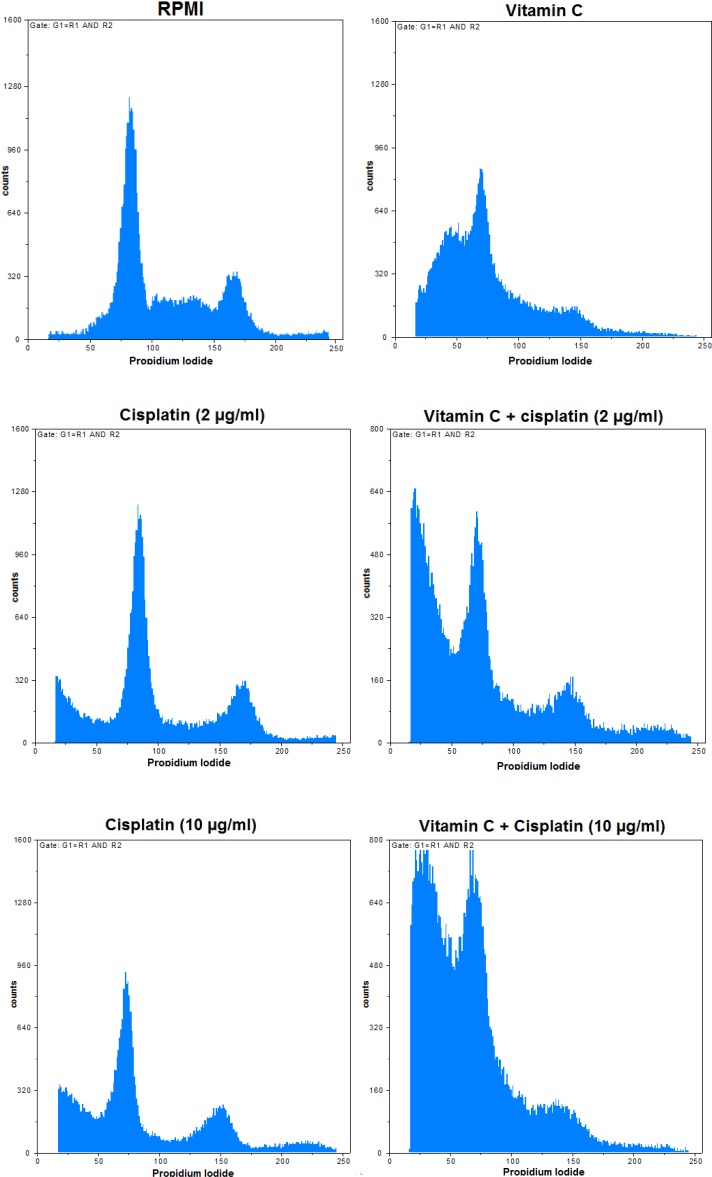
Effects of vitamin C and cisplatin alone and vitamin C + cisplatin treatments on cell cycle distribution of gastric cancer cells. RPMI 1640 was used as the negative control

**Fig. 4:**
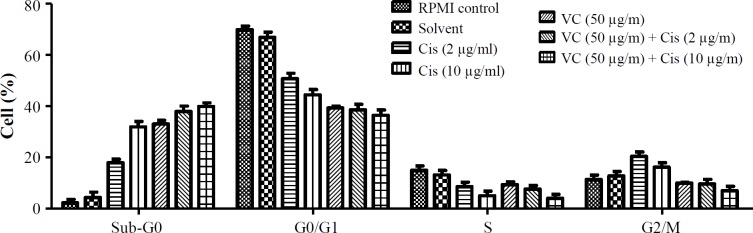
Comparison of vitamin C (VC) and cisplatin (Cis) properties on cell cycle distribution of AGS cells. Data are shown with SD

 In order to determine the DNA count in each cell cycle stage for the AGS cells treated by vitamin C alone and in combination with cisplatin (Fig. 3*)*, PI staining was employed* and the results were compared *(Fig. 4*) based on *p* value calculation (*Table 2*). *Vitamin C alone and in combination with cisplatin (2 and 10 µg/ml) could increase the cell count in the sub-G0 phase, but the single dose of vitamin C could decrease the cell count in the G0/G1 phase, as compared to the RPMI control group (p < 0.05). Meanwhile, vitamin C in combination with cisplatin (2 and 10 µg/ml) could decrease the cell count in both G0/G1 and S phases in *comparison with* RPMI control group (p < 0.05). Furthermore, single and combined doses of vitamin C with cisplatin (2 and 10 µg/ml) could decrease in G2/M phase in *comparison with* cisplatin in single doses (p < 0.05). Vitamin C (50 µg/ml) plus cisplatin (2 and 10 µg/ml) in combination doses could decrease significantly (p < 0.05) the cell count in G0/G1 phase in *comparison with* the single dose of cisplatin (2 µg/ml). Indeed, vitamin C (50 µg/ml) plus cisplatin (10 µg/ml) in combination dose could decrease cell count in G0/G1 in *comparison with* both single doses of cisplatin (2 and 10 µg/ml; p < 0.05). These data clarified that the pointed combination treatments of vitamin C and cisplatin could induce cell deaths in AGS cells in comparison with the single doses of cisplatin and control groups.

**Table 1:** Combination cytotoxic effects of vitamin C with cisplatin on AGS cell line based on the calculation of CI and Fa

**Samples **	**Vitamin C** **dose (µg/ml)**	**Cisplatin ** **dose (µg/ml)**	**CI**	**Fa**
Vitamin C + cisplatin	100	10	0.46620	0.68
	50	10	0.28337	0.64
	100	2	0.47392	0.64
	50	2	0.85337	0.20

Apoptosis and necrosis detection

**Fig. 5:**
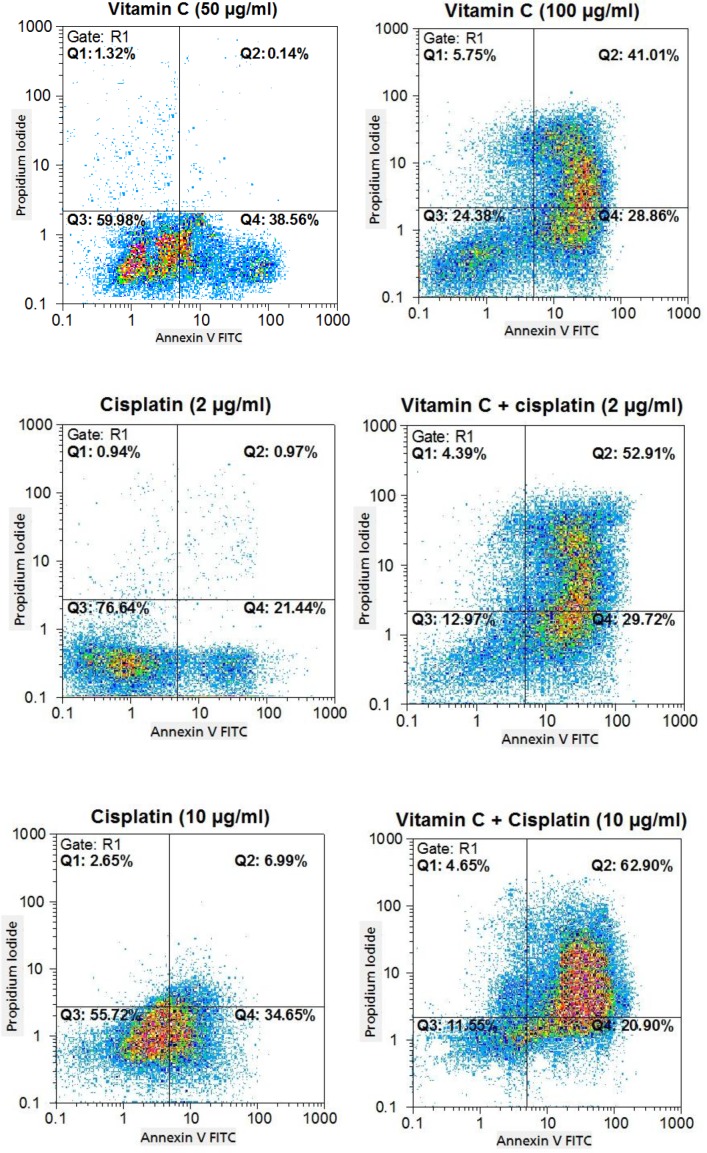
The percentages of apoptotic- and necrotic-treated AGS cells with vitamin C, cisplatin, and vitamin C plus cisplatin, shown in flow cytometry dot plots

**Fig. 6:**
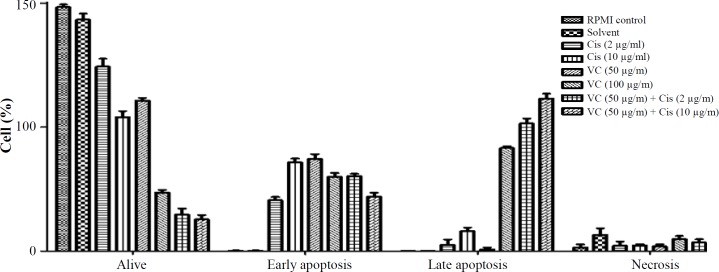
Comparison of cytotoxicity effects of vitamin C (VC) and cisplatin (Cis) properties on cell cycle distribution of AGS cells, data shown with SD

 To study whether the cytotoxicity effects of vitamin C, cisplatin, and vitamin C plus cisplatin on AGS cells were due to the induction of apoptosis, necrosis, or both of them (Fig. 5*)*, the annexin-V and PI double staining method was employed *and the results were compared *(Fig. 6*) based on *p* value calculation (*Table 3*)*. As shown in Figure 6, treatment of cells with vitamin C in single dose (50 µg/ml) led to decreasing alive and increasing early apoptotic cell counts compared with cisplatin (2 µg/ml; p < 0.05). Besides, vitamin C in single dose (50 µg/ml) could decrease cell count in late apoptotic and necrotic phases. Furthermore, vitamin C in the single dose of 100 µg/ml could decrease significantly (p < 0.05) the alive and increase early and late apoptotic cell counts compared with cisplatin of 2 µg/ml. Using vitamin C in single dose (100 µg/ml) led to significant (p < 0.05) decrease in alive and early apoptotic and increase in late apoptotic cell counts compared with cisplatin (10 µg/ml). Finally, the data showed that vitamin C (50 µg/ml) plus cisplatin (2 and 10 µg/ml) in combination manner could reduce alive and induce early and late apoptotic cell counts compared with 2 µg/ml single dose of cisplatin, but in comparison with 10 µg/ml single dose of cisplatin, it decreased and induced early and late apoptotic cell counts, respectively (*p* < 0.05).

**Table 2:** Effects of vitamin C (VC) and cisplatin (Cis) alone and in combination on cell cycle of AGS cells

**Sample**	**Controls**	**Cell cycle (% cells)**					**Cisplatin** (µg/ml)	**Cell cycle (% cells)**			
		**Sub G0**	**G0/G1**	**S**	**G2/M**			**Sub G0**	**G0/G1**	**S**	**G2/M**
VC (50 µg/ml)	RPMI control	*p *< 0.001	*p *< 0.001	Ns	Ns		2	*p *< 0.001	*p *< 0.001	Ns	*p *< 0.001
	Solvent control	*p *< 0.001	*p *< 0.001	Ns	Ns		10	*p *< 0.05	Ns	Ns	*p *< 0.05
											
VC (50 µg/ml) + Cis (2 µg/ml)	RPMI control	*p *< 0.001	*p *< 0.001	*p* < 0.01	Ns		2	*p *< 0.001	*p *< 0.001	Ns	*p *< 0.001
	Solvent control	*p *< 0.001	*p *< 0.001	Ns	Ns		10	*p *< 0.01	Ns	Ns	*p *< 0.05
											
VC (50 µg/ml) + Cis (10 µg/ml)	RPMI control	*p *< 0.001	*p *< 0.001	*p *< 0.001	Ns		2	*p *< 0.001	*p *< 0.001	Ns	*p *< 0.001
	Solvent control	*p *< 0.001	*p *< 0.001	*p *< 0.001	Ns		10	*p *< 0.001	*p *< 0.05	Ns	*p *< 0.01

Ns, non-significant difference (*p *> 0.05);   decreasing and   increasing cell percentage in the cell cycle phases because of cell treatments by samples in comparison with controls and Cis

## DISCUSSION

Cancer and its treatment approaches are the most serious health challenges, and World Health Organization relevant report has estimated that the rate of cancer (the number of new cases of cancer) will be more than 15 million per year in 2020. Nowadays, in spite of the introduction of a wide range of innovative cancer therapy approaches, the chemotherapy-based methods are efficient and common treatment strategies alone and in parallel with surgery and radiotherapy^[^^8^^]^. 

 Cisplatin has been renowned as an anticancer drug with wide usage for chemotherapy of different tumors such as testicular, head and neck, ovarian, cervical, non-small cell lung, and gastric carcinoma. The major mechanism of cytotoxic action of cisplatin is related to its reaction with inter- and intra-strands DNA, and formation of covalent cisplatin-DNA adducts. Formation of cisplatin-DNA adduct leads to a series of enzymatic cascades in nucleus, cytosol, and cell surface in addition to immune responses, which these enzymatic cascades and molecular responses cause cell death. These nonspecific cytotoxic effects of cisplatin imposes wide ranges of side effects on kidney and liver as well as nervous and hearing systems and induce undesirable drug resistance phenomenon^[8]^. To overcome the adverse effects caused by the administration of cisplatin, some combination treatment strategies have been used such as prescribing of cisplatin in combination with WR2721, quercetin, and cordycepin^[^^8^^]^. Additionally, the previous study carried out by our research group showed that herbal extract, including *Morus alba*, *Musa sapientum*, *Arnebia* decumbens*, *Arnebia echioides*, and Arnebia* linearifolia DC could induce the toxicity effect of cisplatin against A2780/cp (human ovarian carcinoma/resistant to cisplatin) cells in parallel with the reduction of its toxicity on HF2 (normal human fetal fibroblast) cell line^[^^13^^]^. Evidence has also suggested the anticancer activity of vitamin C against a variety of cancer cell lines^[2]^. The current investigation was carried out to examine the efficiency of vitamin C in the reduction of the toxicity exposed by cisplatin. Previous investigations have observed that the therapeutic effectiveness of cisplatin was more favorable with vitamin C than its single use^[^^8^^]^. In addition, vitamin C could reduce the systemic toxicity of cisplatin in the mice model, especially with protecting kidney and liver in addition to sperms against cisplatin-induced toxicity and abnormality based on the reduction of glutathione level and induction of lipid peroxidation reactions^[^^14^^]^. A similar study has also identified that vitamin C could decrease nephrotoxicity caused by cisplatin without reduction in the efficacy of cisplatin in C57BL/6 mice with Lewis lung carcinoma^[^^15^^]^. Additional studies have demonstrated that the co-administration of vitamin C and cisplatin could protect kidney and normal cells against cisplatin-induced nephrotoxicity and genotoxicity *in vivo*^[^^15^^-^^17^^]^.

**Table 3:** Comparison of vitamin C (VC) and cisplatin (Cis) properties on inducing apoptosis/necrosis

**Sample **	**Cisplatin** (µg/ml)	**Cell percentage**			
		**Alive**	**Early apoptosis**	**Late apoptosis**	**Necrosis**
VC (50 µg/ml)	2	*p *< 0.001	*p *< 0.001	Ns	Ns
				
	10	Ns	Ns	*p *< 0.01	*p *< 0.01
					
VC (100 µg/ml)	2	*p *< 0.001	*p *< 0.001	*p *< 0.001	Ns
				
	10	*p *< 0.001	*p *< 0.05	*p *< 0.001	Ns
					
VC (50 µg/ml) + Cis (2 µg/ml)	2	*p* < 0.001	*p* < 0.001	*p* < 0.001	Ns
				
	10	*p *< 0.001	*p *< 0.05	*p *< 0.001	Ns
					
VC (50 µg/ml) + Cis (10 µg/ml)	2	*p *< 0.001	*p *< 0.05	*p *< 0.001	Ns
				
	10	*p *< 0.001	*p *< 0.05	*p *< 0.001	Ns

Ns, non-significant difference (*p* > 0.05); , decreasing and increasing alive, early apoptotic, late apoptotic and necrotic cell percentages because of cell treatments by samples in comparison with Cis

Based on previous *in vitro* and *in vivo* investigations, the current research was carried out to present potential approach against gastric cancer based on increasing the efficacy of treatment in addition to decreasing the cisplatin induced systemic toxicity such as hepatotoxicity, nephrotoxicity, and genotoxicity. The results of co-treatment of AGS cells by vitamin C and cisplatin confirmed the synergistic interaction between vitamin C and cisplatin for inducing cytotoxicity against gastric cancer cells *in vitro*. 

 The IC_50_ values for vitamin C (49.38 ± 0.82 µg/ml) and cisplatin (10.58 ± 0.49 µg/ml) in single use revealed the required information for selecting optimal doses for further experiments as combination treatment-based assay, cell cycle, and apoptosis/ necrosis determination analyses. The co-treatment of AGS cells with vitamin C in 50 and 100 µg/ml doses in addition to cisplatin in 10 and 2 µg/ml doses showed that vitamin C could enhance the *in vitro* anticancer effect of cisplatin in both its IC_50 _and five times less than IC_50 _doses in synergistic manner with CIs < 1. 

 In this study, flow cytometry-based methods were performed to determine *apoptotic* and necrotic cell death and also the cell cycle distribution of the treated AGS cells with vitamin C (100 µg/ml) and cisplatin (2 and 10 µg/ml) alone and in combination doses (vitamin C in 50 µg/ml dose plus cisplatin in 2 and 10 µg/ml doses). 

 The results of flow cytometry revealed that vitamin C alone and in combination with cisplatin in two tested doses could elevate the percentage of cells in the sub-G0 phase compared with cisplatin single therapy. Moreover, treatment of cells with vitamin C alone and in combination with cisplatin led to increasing the percentage of apoptotic and necrotic cells, as compared to the negative control and cisplatin single treatment.

 Taken together, the findings of current research show that the co-administration of vitamin C with cisplatin may be developed as an innovative, effective therapeutic strategy for patients with gastric cancer in the future.
